# Medicine and Therapeutics

**Published:** 1898-02

**Authors:** 


### MEDICINE AND THERAPEUTICS.

### TREATMENT OF INTESTINAL CATARRH.

IN CASES of obstinate subacute intestinal catarrh, where simple astringent and digestive agents fail to produce satisfactory improvement, a combination of remedies in accordance with the following prescription (Dr. A. L. Hall, of Fairhaven, N. Y.,) will usually render prompt benefit:

℞ Tr. ferri chloridi ............................ dr. ii.
Tr. catechu ................................... dr. iii.
Tr. Opii camphoratæ ........................ dr. iv.
Ext. rhei aromatic fl. ........................ dr. ii.
Spts. vini gallici ............................ oz. ii.
Syrupi simplicis .................... q. s., ad., oz. viii.

M. Sig.—For a child one to two years old, one-half to one teaspoonful every four to six hours.

### THE DRUG TREATMENT OF INSOMNIA.

Dr. R. Ferguson, lecturer on therapeutics in the Western University (*British Med. Jour.*), presented at the last meeting of the British medical association some practical suggestions on the use of drugs in the treatment of insomnia. While he did not believe that the hypnotic is yet discovered, or ever will be, which is at once trustworthy as to producing the result desired and incapable of producing any unpleasant after-effects, sulfonal, in his opinion, came as near to this standard as any drug with which he was acquainted. The tardiness and permanency of its action was undoubtedly a consequence of its difficult solubility and slow absorption and could not be looked upon as a disadvantage if properly allowed for, since a second good night without a repetition of the dose was by no means an unusual occurrence. In the author's practice it had failed but rarely when given in the dose of 1 gramme. He had rarely, if ever, repeated this dose on the same night. In doses of less than thirty centigrammes it might be properly considered a semi-placebo. It should not be given continuously, that is, on successive nights, for any length of time and if its use has to be continued the intervals should be even longer than two days. The peculiarities of action of sulfonal could be best utilised by reserving it for a patient who had not slept for several nights and who it was pretty clear was not going to get a good night unassisted, so that the medicine may be given early—namely, 6 to 7 P. M., in order that its effect may be developed at about the regular time for going to sleep.

---

ANTIPYRIN FOR ATONIC ULCERS OF THE LEG.—Bosse, of Domnau, (*Berl. klin. Wochenschr.*) says: In a case of hemorrhage from an ulcer of the leg, antipyrin, spread on a piece of cotton wool, was applied. After a few days, on removal of the cotton wool, it was seen that the place was covered with splendid healthy-looking granulations. The author now spread antipyrin over the whole ulcerated surface, which was about the size of the palm of the hand. After ten days this large surface was covered with solid granulations and after twenty-two days the ulcer had completely healed by the aid of an ointment containing 2 per cent. of nitrate of silver. A similarly successful treatment was carried out in twenty-nine other cases.

The application of antipyrin, which was repeated every day, caused considerable pricking and burning pain, which lasted from five to fifteen minutes. This pain disappeared as soon as the drug had become liquified. It was considerably lessened by previous application of cocaine.

Signs of inflammatory reaction were never observed. In all cases where inflammatory signs existed already and the ulcer was very painful, the extremity edematous and the ulcers covered with sloughs and fetid discharge, it was found better to advise absolute rest and to begin the treatment by disinfectant and soothing applications. When all signs of inflammation had disappeared, antipyrin was dispersed over the ulcers. When, after using antipyrin, the granulations have become solid and abundant, treatment with various ointments, especially silver nitrate ointment, is recommended.

